# Zinc Content in Breast Milk and Its Association with Maternal Diet

**DOI:** 10.3390/nu10101438

**Published:** 2018-10-05

**Authors:** Līva Aumeistere, Inga Ciproviča, Dace Zavadska, Konstantīns Bavrins, Anastasija Borisova

**Affiliations:** 1 Faculty of Food Technology, Latvia University of Life Sciences and Technologies, Rīgas iela 22, LV-3004 Jelgava, Latvia; inga.ciprovica@llu.lv; 2 Institute of Food Safety, Animal Health and Environment BIOR, Lejupes iela 3, LV-1076 Riga, Latvia; konstantins.bavrins@bior.lv (K.B.); anastasija.borisova@bior.lv (A.B.); 3 Department of Pediatrics, Riga Stradiņš University, Vienības gatve 45, LV-1004 Riga, Latvia; dace.zavadska@rsu.lv

**Keywords:** breast milk, zinc, lactation, diet

## Abstract

Background: Zinc is an indispensable element, being involved in many biological processes. Correspondingly, insufficient zinc intake in early youth can detrimentally affect the function of a growing body. The aim of this study was to determine zinc content in breast milk among lactating women in Latvia and factors (maternal diet; mother’s and baby’s characteristics; breastfeeding pattern) affecting it. Methods: In total, 62 mature milk (at least one month postpartum) samples were collected and pooled within 24 h. Zinc content (mg 100 mL^−1^) was determined using inductively coupled plasma mass spectrometry (ICP-MS; Agilent 7700×, Agilent Technologies, Tokyo, Japan). Results: Zinc content in mature breast milk ranged from 0.01 to 0.34 mg 100 mL^−1^ with a median (interquartile range) content of 0.10 (0.05–0.15) mg 100 mL^−1^. Time postpartum was a significant negative predictor for zinc content in breast milk (*r* = −0.500; *p* = 0.000). Median maternal zinc intake was 10.70 (7.24–15.27) mg. Yet, zinc content in breast milk was unaffected by maternal dietary zinc intake (*r* = 0.155; *p* = 0.221). Conclusions: Maternal dietary zinc intake was nearly the recommended intake for lactating women (11 mg), but due to low zinc content in breast milk, babies in Latvia might not receive sufficient zinc intake. Future research should aim for the assessment of zinc status by evaluating plasma or serum levels of both mothers and babies.

## 1. Introduction

Zinc is an essential nutrient, particularly in early youth [1]. Zinc impacts the function of the immune system and is needed for normal keratinisation processes. This element also acts as a cofactor for many enzymes [1,2].

The World Health Organization (WHO) appraises that zinc deficiency is a worldwide problem. More than half a million deaths in infants and young children under the age of five years are due to zinc deficiency [3]. The highest percentage of deaths accounted for by diarrhea, malaria, and pneumonia among children are from Latin America, Africa, and Asia [4]. Lower zinc intake in these regions is associated with overall lower energy and animal protein, but higher phytate intake [5].

Breast milk is commonly the sole source of food (and accordingly, the zinc source) for babies for the first six months of life, and mothers are encouraged to continue breastfeeding in addition to complementary feeding until two years of age and longer [6]. Mean fractional absorption of zinc from breast milk is approximately 50%; therefore, the baby’s zinc demand is fulfilled for at least the first several months, although the quantity of zinc transferred from the mammary gland to the breastfed baby decreases as the lactation progresses [7,8]. It is also speculated that zinc is the first limiting nutrient in breast milk, because the decline in zinc amount from colostrum to mature milk is substantial: from about 4 mg per day during the first days postpartum to approximately 0.7 mg per day by six months [7,8,9]. Some studies [10,11,12] indicate that zinc intake from breast milk for babies under six months of age can already be lower than recommended. Nevertheless, the time period during which breast milk alone can provide the necessary zinc amount remains uncertain [8].

The European Food Safety Authority (EFSA) states that current zinc intakes for infants and young children in Europe are mainly above the average requirements and apparent deficiency in this population group has not been reported [13]. However, symptoms of zinc deficiency are nonspecific (failure to thrive, reduced immune response, dermatitis, poor appetite, irritability), and diagnosis of zinc deficiency is also difficult because of the absence of a sensitive biomarker of zinc status, and therefore mild zinc deficiency can pass undetected [1,2,7]. Daily zinc requirements for infants and toddlers are shown in Table 1.

Zinc content in breast milk for at least the first four to six months of lactation is greater than in maternal plasma, but the transport mechanism for zinc secretion in breast milk is not fully understood [16]. Mean fractional absorption of zinc from the diet for females is about 31%, but it can double during lactation in response to zinc excretion via breast milk [7,17]. Zinc-rich food sources are meat, eggs, and fish. Plant-based products such as legumes and grain-based products are a good source of zinc as well, but they also contain phytates, which decrease the bioavailability of dietary zinc [7]. Although women are encouraged to consume more zinc-rich food during lactation, studies have shown that zinc content in breast milk is sustained tightly and is not affected by maternal zinc status, dietary zinc intake, or ingestion of zinc-rich supplements [7,9,10,14,16,18,19].

According to data from The Centre for Disease Prevention and Control of Latvia [20], in 2017, approximately 58% of women in Latvia were breastfeeding for at least six and about 26% for at least 12 months. To reduce zinc loss via breast milk, Ministry of Health of the Republic of Latvia recommends that a woman’s dietary intake of zinc during lactation should be increased: from 7 to 11 mg per day [7,15]. These recommendations were developed in 2017 and are based on Nordic Nutrition Recommendations [21] and guidelines from the EFSA [7]. Previous guidelines [22] suggested much higher daily zinc intakes for nonlactating and lactating women: 14 and 19 mg, respectively. The National Food Consumption Survey of Latvia conducted from 2007 to 2009 is the latest compilation regarding nutrient intake among Latvian citizens [23]. Breastfeeding women were not included in the study, and therefore there is currently no data about zinc intake among lactating women in Latvia, but survey [23] results showed that the average zinc intake for nonlactating women was 7.18 mg per day (value excluding potential zinc intake from dietary supplements), which is adequate, according to current dietary recommendations [15]. Before our preliminary results [24,25], there was also no data about zinc content in milk among breastfeeding women in Latvia. The aim of the study was to determine zinc content in breast milk and factors (maternal diet, mother’s and baby’s characteristics, and breastfeeding method) affecting it.

## 2. Materials and Methods

A poster with an invitation to take part in this cross-sectional study was published on a social media member group for breastfeeding mothers.

To obtain the necessary number of samples for this pilot study, we used an online calculator [26]. With a probability of 0.05 and confidence level of 95%, we calculated that at least 59 samples were needed [27].

In total, 66 women were recruited from November 2016 until December 2017. The inclusion criteria for participants were:

1. currently living in Latvia;
2. singleton pregnancy;
3. baby’s birth weight above 2.50 kg;
4. at least one month postpartum;
5. currently exclusively breastfeeding or partially breastfeeding (breast milk + infant formula and/or complementary food);
6. currently breastfeeding only one child;
7. mother and baby currently in good health (without metabolic disorders, no acute illnesses, etc.).

The study was conducted in accordance with the Declaration of Helsinki, and the protocol was approved by the Riga Stradiņš University Ethics Committee (No. 4/28.7.2016.). Written informed consent was obtained from all women before the participation in the study. Exclusion criteria were: noncompliance with the inclusion criteria; unsigned consent form.

Self-administered food dietary records on the day before milk sampling were obtained (24-h food diary). No dietary recommendations were given before the study. Size measures (spoons, etc.) and a photographic atlas of food portions [28] were used to help complete the food diary. A self-administered semistructured food frequency questionnaire (FFQ) was used to assess the consumption frequency of 74 commonly consumed food items over the month prior to the study. Food items in the FFQ were combined into comprehensive food groups: grain-based products, eggs, meat, fish and seafood, milk and milk products, vegetables and legumes, fruit and berries, nuts, seeds, vegetable oils and shortenings, condiments, sweets and snacks, sweetened carbonated drinks, caffeine-containing drinks, herb teas, and alcohol.

The response options were arranged in six categories, from “never” (0 points), “less than once a week” (1 point), “once a week” (2 points), “twice a week” (3 points), “more than twice a week but not every day” (4 points), to “every day” (5 points). The FFQ was adapted from guidelines developed by the World Health Organization [29].

A qualified nutritionist inspected both the food records and FFQ to ensure that they were complete and satisfactory. Total dietary zinc intake was estimated using data from the USDA Branded Food Products Database [30]. Data about the zinc content of different dietary supplements was taken from the manufacturers’ websites.

Information about characteristics such as maternal age, parity, time postpartum, sex and birth weight of the baby, and breastfeeding pattern (exclusive or partial) was also collected.

Four out of 66 participants at this stage were excluded from the study because they did not complete the food diary, FFQ, and/or information about characteristics. Mothers were instructed to collect breast milk at home and to obtain a pooled sample (approximately 10 mL) within a 24-h period. To diminish the disruption (nutritional or behavioral) to the mother and baby, only hindmilk was obtained, by expressing a few milliliters of milk after the end of nursing from the feeding breast. Sampling frequency was not defined, but the pooled sample had to include milk from the morning, mid-day, and evening feedings. To minimize discomfort, women could use the most convenient milk expression method. The sample was collected into a prelabelled polypropylene container which was stored in the refrigerator (4 ± 2 °C) during the collection process, and after that, placed in the household freezer (15–18 °C). Samples were transported to the laboratory in a cooler with ice packs and kept frozen at −18 ± 3 °C until the analysis. The measurements were performed according to the ICP-MS Agilent 7700× manufacturer’s instructions. Zinc (^66^Zn) content in all breast milk samples was analyzed in duplicate. Blank and quality-control samples were used to monitor the efficiency and accuracy of ICP-MS analysis. The results from ICP-MS were expressed as mg 100 mg^−1^. Breast milk density (1.03 g mL^−1^) was used [31] to convert results to mg 100 mL^−1^ so that our data could be comparable with other studies.

All data were summarized using Microsoft Excel, 2016 (Microsoft Corp, Redmond, WA, USA), and statistical analyses were performed using IBM SPSS Statistics, version 22.0 (SPSS Inc., Chicago, IL, USA). The results were expressed as median (interquartile range), mean ± standard deviation, or minimal–maximal values. Spearman’s correlation coefficient was used to evaluate association among continuous variables. The Mann–Whitney U test or Kruskal–Wallis test was used for categorical variables. A *p*-value of less than 0.05 was considered as statistically significant.

## 3. Results

### 3.1. Characteristics of Participants

Mean age for participants (*n* = 62) was 31 ± 4 years. Primiparas comprised 24 participants. Age for babies (33 males, 29 females) ranged from 1.5 to 16.0 (5.5 ± 3.5) months and the mean birth weight was 3.54 ± 0.47 kg, respectively. Most mothers (*n* = 35) were exclusively breastfeeding during the study period; two mothers were combining breastfeeding with formula feeding, but 25 mothers’ babies were receiving breast milk along with complementary foods.

### 3.2. Zinc Content in Breast Milk

**Figure 1:**
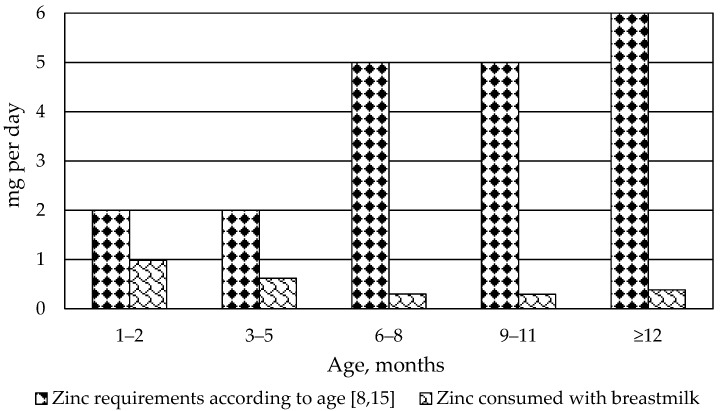
Median amount of zinc transferred with breast milk, depending on the baby’s age.

Zinc content in mature breast milk (*n* = 62) ranged from 0.01 to 0.34 mg 100 mL^−1^, with a median content of 0.10 (0.05–0.15) mg 100 mL^−1^. Total zinc amount transferred with breast milk (Figure 1) was estimated using our obtained results and data from the World Health Organization Programme of Nutrition about the intake of breast milk for babies from industrialized countries [3].

### 3.3. Maternal Diet during Lactation and Zinc Content in Breast Milk

Energy value and nutrient intake by participants are shown in Table 2. Overall, maternal zinc intake on the day before milk sampling ranged from 4.97 to 49.99 mg, with a median intake of 10.70 (7.24–15.27) mg. Neither maternal zinc intake (*r* = −0.155, *p* = 0.230) nor other nutrient intake correlated with zinc content in breast milk (*p* > 0.05 for all). Ten of the participants indicated the use of dietary supplements containing zinc on the food diary, but it did not significantly affect zinc content in breast milk (*p* = 0.991).

Table 3 summarizes the data about the most commonly consumed food groups among participants (median consumption frequency ≥ 7 points), which also included zinc-rich products (meat, legumes, grain-based products). However, data from the FFQ did not reveal any significant influence on zinc content in breast milk (*p* > 0.05 for all food items and groups).

### 3.4. Association among Characteristics of Participants and Zinc Content in Breast Milk

Time postpartum was a significant negative predictor for zinc content in breast milk (*r* = −0.500; *p* = 0.000). This relation was still significant when results were divided into two groups, depending on time postpartum (Table 4). Breast milk from exclusively breastfeeding mothers contained significantly higher zinc content that breast milk from mothers who were partially breastfeeding (Table 5). However, after conducting a partial correlation between zinc content in breast milk and breastfeeding pattern while controlling for time postpartum, the correlation (*r* = −0.105) was no longer significant (*p* = 0.421). Other characteristics did not significantly influence zinc content in breast milk (Table 6).

## 4. Discussion

Breastfeeding rates in Latvia are quite high; more than half of the mothers in Latvia (58%) are breastfeeding for at least the first six months and about 26% of women for at least the first 12 months [20]. However, it is difficult to assess if Latvian babies’ demands for zinc via breastmilk are fulfilled, as currently there is only preliminary data about zinc content in breast milk among women from Latvia [24,25].

Our obtained median zinc content (0.10 mg 100 mL^−1^) in breast milk was lower compared to data from Poland (0.16 mg 100 mL^−1^) [10]. Similar zinc content (approximately 0.11 mg 100 mL^−1^) was detected in mature breast milk samples among lactating women from Portugal [32]. Lower zinc content (0.07 and 0.05 mg 100 mL^−1^) has only been reported by Domellöf et al. [33], for breast milk samples from mothers in Honduras and Sweden, respectively. Overall, it is difficult to compare data between studies due to differences in sampling plans, time postpartum, and analytical methods used.

Our obtained lower zinc results could be explained by the fact that we collected only hindmilk samples. Doneray et al. [34] observed that zinc content in hindmilk was approximately 0.2 mg lower than in foremilk; however, other researchers [35,36] have not observed variations in zinc content between fore- and hindmilk. Alam et al. [37] identified that mutation in the protein ZnT2 (SLC30A2) that transports zinc can lead to low content of this element in breast milk (<0.09 mg 100 mL^−1^), but further research is needed.

Maternal zinc intake in our study (10.70 mg per day) was nearly the daily intake proposed for lactating women by the Ministry of Health of the Republic of Latvia: 11 mg per day [15]. Nevertheless, the majority of studies has pointed out that maternal dietary zinc intake does not affect zinc content in breast milk [38,39], which is in agreement with our results.

One factor that influences zinc content in breast milk is the time postpartum [7]. This observation is well documented in studies from other countries [9,10,40,41] and was also noted in our study. Prolactin may be involved in the secretion of zinc from the mammary gland; the circulating prolactin level declines as lactation proceeds, and accordingly, zinc content in breast milk decreases as lactation proceeds [18,42].

Zinc-deficiency symptoms are nonspecific and therefore mild zinc deficiency can pass undetected [1]. One of the inclusion criteria for our study was that participating mothers and their babies were currently in good health, which indicates that no evident zinc deficiency was showing for the babies; however, according to our results, babies until six months of age in Latvia might not receive sufficient zinc intake with breast milk. Further research should aim to assess babies’ plasma or serum levels, as there is some evidence that babies may be able to acquire a portion of their zinc needs by mobilizing hepatic reserves accumulated during gestation [43].

We observed significantly lower zinc content in breast milk after six months of lactation, but this is also the period when complementary feeding should be started [6]. A median intake of zinc from complementary foods is about 1.48 mg per day [14]. Summarizing those data with our obtained results, we conclude that due to low zinc content in breast milk, babies after six months of age could also receive less zinc than recommended, but it depends on the quality of the complementary feeding. At higher risk for zinc deficiency are those breastfed babies that primarily receive plant-based complementary foods [5]. Introduction of meat—a good source of zinc—as an early complementary food is practicable and associated with increased zinc intake [44]. Current information about food patterns among babies in Latvia states that after six months of age, 73% of babies are consuming meat as a complementary food on a daily basis [45], but further research is needed to calculate the actual zinc intake for Latvian babies who had started weaning.

## 5. Conclusions

Babies in Latvia might not receive sufficient zinc intake with breast milk. Zinc content in breast milk was affected by time postpartum. Maternal dietary zinc intake was nearly the recommended intake for lactating women. Future research should aim for the assessment of zinc status by evaluating plasma or serum levels of both mothers and babies.

## Figures and Tables

**Table 1:** Daily zinc intake requirements for infants and toddlers.

Age (months)	Daily Zinc Intake (mg)
0–6	2.0 ^1^
7–12	3.0 1–5.0 ^2^
11–36	3.0 1–6.0 ^2^

^1^ Institute of Medicine (United States) [14]. ^2^ Ministry of Health of the Republic of Latvia [15].

**Table 2:** Energy value and nutrient intake by participants and correlation with zinc content in breast milk (*n* = 62).

Nutrient (unit)	Median Value (Interquartile Range)	Spearman Correlation Coefficient *r* (*p* Value)
Energy (kcal)	2095.00 (1785.75–2564.50)	*r* = 0.042 (*p* = 0.746)
Energy (kJ)	8775.10 (7464.44–10,719.61)	*r* = 0.042 (*p* = 0.746)
Protein (g)	80.77 (55.43–109.26)	*r* = −0.088 (*p* = 0.499)
Total lipid (g)	91.63 (62.89–126.83)	*r* = −0.013 (*p* = 0.922)
Carbohydrate (g)	253.34 (194.27–300.70)	*r* = 0.138 (*p* = 0.286)
Fibre, total dietary (g)	28.90 (19.00–37.18)	*r* = −0.041 (*p* = 0.750)
Sugars (g)	106.23 (78.45–133.77)	*r* = 0.055 (*p* = 0.671)
Zinc (mg)	10.70 (7.24–15.27)	*r* = −0.155 (*p* = 0.230)

**Table 3:** Food intake frequencies and correlation with zinc content in breast milk (*n* = 62).

Food Item or Group	Median (Min–Max) Value of the Consumption Frequency ^1^	Spearman Correlation Coefficient *r* (*p* Value)
Grain-based products (16 food items included)	20 (0–35)	*r* = 0.008 (*p* = 0.953)
Meat (4 food items included)	7 (0–15)	*r* = −0.074 (*p* = 0.566)
Milk and milk products (7 food items included)	19 (0–29)	*r* = −0.099 (*p* = 0.446)
Vegetables and Legumes (10 food items included)	20 (10–30)	*r* = −0.212 (*p* = 0.098)
Fruits and berries (7 food items included)	14 (0–26)	*r* = −0.218 (*p* = 0.089)
Vegetable oils and shortenings (4 food items included)	8 (2–14)	*r* = −0.190 (*p* = 0.139)
Sweets and snacks (10 food items included)	13 (0–22)	*r* = 0.015 (*p* = 0.907)
Caffeine-containing drinks (3 food items included)	8 (0–15)	*r* = −0.014 (*p* = 0.916)

^1^ The response options were arranged in six categories, from “never” (0 points), “less than once a week” (1 point), “once a week” (2 points), “twice a week” (3 points), “more than twice a week but not every day” (4 points), to “every day” (5 points).

**Table 4:** Time postpartum and zinc content in breast milk (*n* = 62).

Time Postpartum	Zinc Content in Breast Milk	*p* Value
<6 months (*n* = 37)	0.14 (0.08–0.17) mg 100 mL^−1^	0.001
≥6 months (*n* = 25)	0.06 (0.05–0.10) mg 100 mL^−1^	

**Table 5:** Breastfeeding pattern and zinc content in breast milk (*n* = 62).

Breastfeeding Pattern	Zinc Content in Breast Milk	*p* Value
Exclusive breastfeeding (*n* = 35)	0.13 (0.09–0.17) mg 100 mL^−1^	0.001
Partial breastfeeding (*n* = 27)	0.06 (0.05–0.11) mg 100 mL^−1^	

**Table 6:** Association among characteristics of participants and zinc content in breast milk (*n* = 62).

Characteristic	Spearman Coefficient for Continuous Variables	*p* Value
Time postpartum	*r* = −0.492	0.000
Maternal age	*r* = −0.178	0.167
Parity	*r* = 0.190	0.137
Baby’s birth weight	*r* = −0.167	0.194
Baby’s sex	not applicable	0.589
